# Supplementary material for: Asynchronous magnetic resonance elastography: Shear wave speed reconstruction using noise correlation of incoherent waves
Source: Magn Reson Med. 2022 Oct 27;89(3):990–1001. doi: 10.1002/mrm.29502 (PMC9792433; doi:10.1002/mrm.29502)
Supplement: Supplementary file 1 — DATA S1 MATLAB code used to generate the numerical simulations of Figure 1 [file MRM-89-990-s002.zip › k-Wave/helpfiles/example_pr_2D_tr_autofocus.html]

Automatic Sound Speed Selection Example (k-Wave)


# Automatic Sound Speed Selection Example

This example illustrates an autofocus approach for automatically selecting the sound speed used in photoacoustic image reconstruction. The selection is based on maximising the sharpness of the reconstructed image as quantified by a focus function.

- Open the file in the MATLAB Editor
- Run the file in MATLAB

For a more detailed discussion of this example and the underlying techniques, see B. E. Treeby, T. K. Varslot, E. Z. Zhang, J. G. Laufer, and P. C. Beard, "Automatic sound speed selection in photoacoustic image reconstruction using an autofocus approach," *J. Biomed. Optics*, vol. 16, no. 9, p. 090501, 2011.

## Contents

- Selecting a focus function
- Running the reconstruction

## Selecting a focus function

The reconstruction of images in photoacoustic tomography is reliant on specifying the speed of sound within the propagation medium. However, for in vivo imaging, this value is not normally accurately known. To overcome this, an optimisation of the selected sound speed is performed to maximize the sharpness of prominent features visible within the reconstructed image. This is based on an autofocus approach in which a focus function is used to give quantitative assessment of the image sharpness.

There is a large body of literature concerning the quantification of image sharpness. In general, a sharp image will contain more high-frequency information than its blurry counterpart, however, there is no universally accepted measure for this difference. A variety of sharpness metrics (or equivalently, focus functions) have been proposed, including those based on the image gradient, pixel count, power, variance, entropy, and autocorrelation. The best functions produce a single maximum with a sharp peak when the image is in focus, have a large range between the local minima on either side of the maximum, and are robust to noise in the image.

Here, the image sharpness is estimated using the function `sharpness`. This implements three focus functions that generally perform well: the Brenner gradient, the Tenenbaum gradient, and the normalised variance.

## Running the reconstruction

In this example, the sensor data is generated using a 2D vessel phantom and a full-view circular sensor mask. The selection of the sound speed that maximises the image sharpness is then performed by iteratively reconstructing the image and evaluating the image sharpness.

```
% run the reconstruction in a loop using different values for sound speed
sound_speed_array = 1450:5:1550;
for index = 1:length(sound_speed_array)
    
    % update value of sound speed
    medium.sound_speed = sound_speed_array(index);
    
    % run the reconstruction
    p0_recon = kspaceFirstOrder2D(kgrid, medium, source, sensor, input_args{:});
    
    % update the value of the focus function
    focus_func(1, index) = sharpness(p0_recon, 'Brenner');
    focus_func(2, index) = sharpness(p0_recon, 'Tenenbaum');
    focus_func(3, index) = sharpness(p0_recon, 'NormVariance');
    
end
```

The variation in the focus functions with sound speed is shown below. Each of the three metrics give the same maximum value at 1500 m/s, which corresponds to the actual sound speed in this example.

In general, the efficacy of this approach will depend on both the intrinsic content of the reconstructed image in addition to any image artifacts (for example, limited-view artifacts). If the reconstructed image is rich in features and any image artifacts are comparatively small in magnitude, then the autofocus approach will work correctly to maximize the sharpness of the image features. However, if the converse is true, then the autofocus approach may incorrectly attempt to maximize the sharpness or the prevalence of edges in the image artifacts.
